# Identification and expression profiles of neuropeptides and their G protein-coupled receptors in the rice stem borer *Chilo suppressalis*

**DOI:** 10.1038/srep28976

**Published:** 2016-06-29

**Authors:** Gang Xu, Gui-Xiang Gu, Zi-Wen Teng, Shun-Fan Wu, Jia Huang, Qi-Sheng Song, Gong-Yin Ye, Qi Fang

**Affiliations:** 1 State Key Laboratory of Rice Biology & Key Laboratory of Agricultural Entomology of Ministry of Agriculture, Institute of Insect Sciences, Zhejiang University, Hangzhou 310058, China; 2 College of Plant Protection, State & Local Joint Engineering Research Center of Green Pesticide Invention and Application, Nanjing Agricultural University, Nanjing 210095, China; 3 Division of Plant Sciences, Missouri University, Columbia, MO 65211, USA

## Abstract

In insects, neuropeptides play important roles in the regulation of multiple physiological processes by binding to their corresponding receptors, which are primarily G protein-coupled receptors (GPCRs). The genes encoding neuropeptides and their associated GPCRs in the rice stem borer *Chilo suppressalis* were identified by a transcriptomic analysis and were used to identify potential targets for the disruption of physiological processes and the protection of crops. Forty-three candidate genes were found to encode the neuropeptide precursors for all known insect neuropeptides except for arginine-vasopressin-like peptide (AVLP), CNMamide, neuropeptide-like precursors 2-4 (NPLP2-4), and proctolin. In addition, novel alternative splicing variants of three neuropeptide genes (allatostatin CC, CCHamide 1, and short neuropeptide F) are reported for the first time, and 51 putative neuropeptide GPCRs were identified. Phylogenetic analyses demonstrated that 44 of these GPCRs belong to the A-family (or rhodopsin-like), 5 belong to the B-family (or secretin-like), and 2 are leucine-rich repeat-containing GPCRs. These GPCRs and their likely ligands were also described. qRT-PCR analyses revealed the expression profiles of the neuropeptide precursors and GPCR genes in various tissues of *C. suppressalis*. Our study provides fundamental information that may further our understanding of neuropeptidergic signaling systems in Lepidoptera and aid in the design of peptidomimetics, pseudopeptides or small molecules capable of disrupting the physiological processes regulated by these signaling molecules and their receptors.

Neuropeptides play an important role in the regulation of the development, reproduction, feeding, courtship, aggression, olfaction, locomotor activity, circadian rhythm, and many other physiological processes in insects^1^. Biologically active neuropeptides are generated by large precursors that are cleaved and further modified to produce mature peptides^2^. In insects, mature peptides are secreted into the extracellular environment, where they perform their physiological roles by binding to corresponding receptors, primarily G protein-coupled receptors (GPCRs), which are a large superfamily of proteins characterized by the presence of seven transmembrane domains^3^. Neuropeptide GPCRs belong to the A-family (or rhodopsin-like), the B-family (or secretin-like), or leucine-rich repeat-containing GPCRs (LGRs)^4^. Recent advances in genomic and transcriptomic analyses have led to the continual discovery of neuropeptides and their putative GPCRs in insects, and many insect neuropeptide GPCRs have been deorphanized through pharmacological screening^5^.

The rice stem borer *Chilo suppressalis* (Walker) is one of the most economically important rice pests in Asia, northern Africa, and southern Europe. This insect causes serious crop losses every year, particularly in China because of the prevalence of rice cultivation and the popularization of hybrid varieties^6^. *C. suppressalis* is a lepidopteran pest that represents a diverse and important group of agricultural insect pests that cause widespread economic damage to food and fiber crop plants, fruit trees, forests, and stored grains^7^,^8^,^9^. Currently, chemical pesticides represent the primary method of controlling insect pests, and the excessive use of these chemicals in the field has led to the development of insecticide resistance^10^. Thus, the development of alternative control agents is necessary to resolve this problem. Insect neuropeptides and their GPCRs are promising targets for a novel generation of insecticides that can offer improved specificity and environmental compatibility^11^. Knowledge of neuropeptidergic signaling systems provides fundamental information required for the design of peptidomimetics, pseudopeptides or small molecules capable of disrupting the physiological processes regulated by these signaling molecules and their receptors^2^. Indeed, the GPCR family has been successfully exploited and serves as the target of approximately 26% of all modern medicinal drugs^12^. Thus, the structural and functional characterization of neuropeptidomes from insect pests is the first requirement for developing strategies to replace or complement traditional neurotoxic insecticides^2^.

With the development of next-generation sequencing technology, RNA sequencing (RNA-seq) has become a useful tool for defining the transcriptomes of organisms, even when a reference genome is not available^13^. To obtain a whole transcriptome of neuropeptides and their GPCRs from *C. suppressalis*, we used an RNA-seq approach using Illumina HiSeq technology. In this study, we identified 43 neuropeptide genes and 51 GPCR genes for the neuropeptides of *C. suppressalis* by using query orthologous sequences from *Bombyx mori* and *Drosophila melanogaster* with cDNA cloning, and their expression profiles were confirmed via qRT-PCR. These results allowed us to compare the neuropeptidergic signaling systems in different insect species and provide relevant information for further functional studies in *C. suppressalis*. Although neuropeptides and their putative GPCRs have been reported in the silkworm *B. mori*^7^,^14^, their identification in *C. suppressalis* would further contribute to our understanding of neuropeptidergic signaling systems in Lepidoptera.

## Results and Discussion

### Neuropeptide and peptide hormone genes

RNA-seq data were generated using a central nervous system (CNS) cDNA library and Illumina HiSeq 2000 technology. We acquired 142,051,094 bp raw reads for *C. suppressalis*. After eliminating adapters, ambiguous nucleotides and low quality sequences, 138,063,130 bp clean reads remained. The total clean base pairs yielded 105,769 transcripts and 54,411 unigenes were obtained after assembling the transcripts into unigenes^15^. We identified 43 neuropeptide precursor genes in *C. suppressalis* with a local BLAST search (Table 1). The identified genes included all 38 previously identified neuropeptide genes in *B. mori*^7^. Additional neuropeptide genes were confirmed in *B. mori*, such as allatostatin double C (AstCC)^16^, CCHamide (CCH1)^17^, trissin (TR)^18^, natalisin (NTL)^19^, and RYamide (RY)^4^. Compared with the number of precursor genes in *B. mori*^7^ (Lepidoptera), *D. melanogaster*^20^ (Diptera), *Nilaparvata lugens*^4^ (Hemiptera), *Apis mellifera*^21^ (Hymenoptera), and *Tribolium castaneum*^22^ (Coleoptera), *C. suppressalis* (Lepidoptera) has the second-largest number of neuropeptide precursor genes, and *C. suppressalis* and *B. mori* have similar neuropeptidergic signaling systems (Table 2).

**Figure 1:**
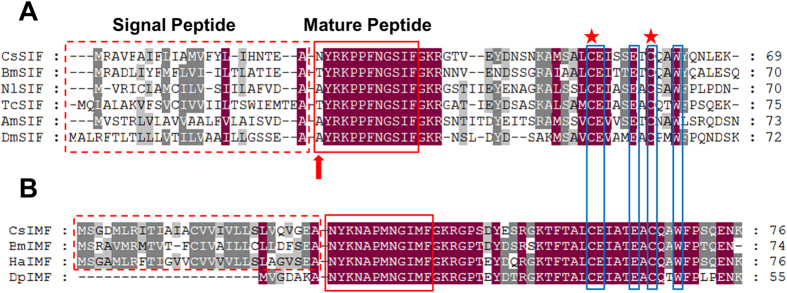
Protein alignment of the SIFamide (**A**) and IMFamide (**B**) precursor sequences from *C. suppressalis* (Cs), *B. mori* (Bm), *N. lugens* (Nl), *T. castaneum* (Tc), *A. mellifera* (Am), *D. melanogaster* (Dm), *H. armigera* (Ha), and *D. plexippus* (Dp). Identities are highlighted in dark red, and similarities are indicated by gray. The dashed boxes indicate the signal peptides, the solid red boxes indicate the mature peptides, and the solid blue boxes indicate the conserved residues between SIFamide and IMFamide. The red asterisks mark the conserved cysteine residues, and the red arrow indicates the one substitution at the N-terminal of SIFamide.

IMFamide is a unique Lepidoptera-specific neuropeptide that has not been identified in other insects (Table 2). The nomenclature for these peptides is based on the three shared C-terminal IMFamide amino acid residues (NYKNAPMNGIMFamide). IMFamide is a highly conserved neuropeptide that is identical in different Lepidoptera species (Fig. 1). In addition, another gene encoding the IMFamide paralogue that contains the C-terminal SIFamide sequence was also identified in *C. suppressalis.* SIFamide is one of the most highly conserved neuropeptides in insects, and it has only one substitution at the N-terminal amino acid (Fig. 1). IMFamide is similar to SIFamide, and the region between two cysteine residues separated by six amino acid residues downstream of SIFamide is particularly striking (Fig. 1). A previous study indicated that IMFamide is generated by the duplication of the SIFamide gene and suggested that the two neuropeptides have a common origin^7^.

The novel neuropeptide NTL, which was named for its function in promoting reproduction (from the Latin *natalis* for “birth”), was recently discovered and characterized in *D. melanogaster*, *T. castaneum*, and *B. mori*^19^. NTL is an arthropod-specific neuropeptide gene encoding multiple copies of mature peptides that contain the C-terminal motif FXXXRa (Supplementary Fig. S1), which is closely related to the motif of tachykinin-related peptides (TKRPs), therefore, NTL is also a tachykinin-like signaling system. NTL modulates sexual activity and fecundity based on the RNAi phenotype in *D. melanogaster* and *T. castaneum*^19^. Thirteen peptides carry the FXXXRa motif, and one peptide contains the YXXXRa motif in the predicted NTL precursor of *C. suppressalis* (Supplementary Fig. S1).

The first periviscerokinin (PVK) was isolated from the perisympathetic organs of the American cockroach *Periplaneta americana* based on myotropic activity^23^. In *D. melanogaster*, a PVK/CAP2b-like peptide was isolated and a gene was later identified and named *capability* (CAPA). The *CAPA* precursor encodes three types of peptides: two PVK-like peptides (CAPA-PVK1 and CAPA-PVK2), a pyrokinin (PK)-like peptide (CAPA-PK, also known as PK1), and the CAPA precursor peptide B (CPPB)^24^ (Supplementary Fig. S2). The CAPA-PVK peptides have a C-terminal AFPRVamide and PKs have FXPRLamide motifs, whereas CAPA-PVK1 has a C-terminal PFPRVamide motif in *B.mori* and *C. suppressalis* (Supplementary Fig. S2). Two neuropeptides, diapause hormone (DH) and pheromone biosynthesis activating neuropeptide (PBAN), are encoded by a single mRNA that also encodes three additional neuropeptides (α-, β- and γ-SGNP (subesophageal ganglion neuropeptide)), which all possess the FXPRLamide motif (Supplementary Fig. S3), and are widespread among moths as well as in other invertebrate species^25^. In *D. melanogaster*, FXPRLamides are located on two different genes, namely the *capa* gene and the *hugin* gene, which contain two peptides CAPA-PK (PK1) and hug-PK (PK2) are the members of the DH/PBAN/PK family (Supplementary Fig. S2).

Ion transport peptide (ITP) was first isolated from the locust *Schistocerca gregaria* based on its antidiuretic activity in the ileum^26^. ITPs is released from the corpora cardiaca and stimulates the ileum to transport Cl^−^ ions from the lumen to the hemolymph, thus forming an electrochemical gradient that drives water resorption^27^. ITP has an alternatively splicing variant named ITP-like (ITPL), which is distinguished by a lack of C-terminal amidation that occurs in ITP (Supplementary Fig. S4). Six spatially conserved cysteines were found in the mature peptide of ITP and ITPL, and these cysteines form three intramolecular disulfide bonds that stabilize the hormone (Supplementary Fig. S5)^28^,^29^. In *C. suppressalis*, ITP encodes 112 amino acids and ITPL encodes 116 amino acids, and they are similar to the ITP of *M. sexta* (AAY29657.1, BLAST E-value 7e-56) and the ITPL of *M. sexta* (AAY29658.1, BLAST E-value 7e-58), respectively (Table 1). ITPs are members of a large neuropeptide superfamily designated as the crustacean hyperglycemic hormone (CHH)^27^.

### Previously unreported splicing variants of three neuropeptide genes

Alternative splicing occurs among different structural and functional gene products. Allatotropin (AT), neuropeptide F1 (NPF1), ion transport peptide (ITP), corticotrophin-releasing factor-like diuretic hormone (CRF-DH), CAPA and orcokinin (OK) transcripts have been reported to undergo differential expression of alternatively splicing products in Lepidoptera^7^. In this study, we reported the splicing variants of three neuropeptide genes.

AstCC is an allatostatin C (AstC)-like peptide and encodes a PISCF-related peptide^16^ (Supplementary Fig. S6). AstCC and AstC are similar peptides, and the two genes were likely generated by gene duplication, and their receptor genes likely have a common ancestor as well^16^. However, the AstCC gene is not a classical neuropeptide gene, and it may well be expressed in cells that do not contain the regulated secretory pathway^16^. In this study, AstCC was found to have two splicing variants: AstCCa, which encodes 140 amino acids; and AstCCb, which encodes 106 amino acids (Supplementary Fig. S7A).

CCH was first identified as CCH2 in *B. mori*^7^, and it is 13 amino acid residues long and contains two cysteines (forming a cystine bond) and a C-terminal histidine-amide group^17^. CCHamide regulates feeding motivation in blowflies^30^ and sensory perception and olfactory behavior in starved *Drosophila*^31^. All insects with sequenced genomes contain two CCH genes, and they each code for a specific CCH: CCH1 (hallmark sequence: SCHSYGHSCWGAHamide), and CCH2 (hallmark sequence: GCQ [or A, or S] AFGHSCY [or F] GGHamide) (Supplementary Fig. S8)^32^. In *C. suppressalis*, two CCHamide genes (CCH1 and CCH2) have been identified. Additionally, in *C. suppressalis*, CCH1 has two splicing variants: CCH1a, which encodes 187 amino acids; and CCH1b, which encodes 162 amino acids (Supplementary Fig. S7B).

Short neuropeptide F (sNPF) was first isolated from the horse shoe crab *Limulus polyphemus*^33^, and the first insect sNPF was identified from the midgut of the cockroach *P. americana*^34^. Because of its length, these neuropeptides are referred to as sNPF to distinguish them from the so-called long neuropeptide F (NPF). sNPF peptides are cleaved from a larger prepropeptide precursor that typically yields multiple sNPF isoforms. In *D. melanogaster*, the sNPF precursor encodes four mature peptides, whereas the sNPF prepropeptide of *A. mellifera* produces only one sNPF isoform^35^. We identified three mature peptides produced from the sNPF precursor of *C. suppressalis*: SVRSPSRRLRFamide, ESRTPVRLRFamide and APSMRLRFamide (Supplementary Fig. S9). sNPF can regulate food intake, body size, growth, insulin production, sleep, and olfactory memory in insects^1^. Here, sNPFa (219 amino acids) and sNPFb (175 amino acids) were identified in *C. suppressalis* (Supplementary Fig. S7C). Interestingly, sNPFa yields one more mature peptide, DARSPVRLRYamide (Supplementary Fig. S7C).

### Lost neuropeptide genes in *C. suppressalis*

In this study, the amino acid sequences of the neuropeptide precursors of *B. mori*, *D. melanogaster* and other insects were used as queries for a local BLAST analysis to search for candidate sequences of neuropeptides from our *C. suppressalis* transcriptomic data. However, we failed to find some neuropeptide precursors (Table 1).

Arginine-vasopressin-like peptide (AVLP) was initially isolated from the CNS of *L. migratoria*^36^, and it has also been identified in *T. castaneum*, *Nasonia vitripennis*, social ants, and *N. lugens*^4^. In this study, we could not find a sequence encoding AVLP in *C. suppressalis.*

CNMamide is a novel insect neuropeptide that was recently discovered in *D. melanogaster* and named after its C-terminal consensus motif^37^. Although CNMamide is conserved in most arthropods, Lepidoptera lack the CNMamide gene. However, the CNMamide receptor (CNMaR) occurs in certain lepidopteran species, including *B. mori* and *Danaus plexippus* (neuropeptide receptor A18)^37^, and CNMaR also occurs in *C. suppressalis.* A recent study also revealed that CNMaR in *B. mori* only shows a weak sensitivity to CNMamide, suggesting that CNMaR has evolved as a receptor for another unknown ligand in *B. mori*^37^.

Neuropeptide-like precursor 2 (NPLP2), NPLP3, and NPLP4 have been identified in *D. melanogaster*^38^, NPLP2 and NPLP3 have been identified in *A. mellifera*^21^, and NPLP3 and NPLP4 have been identified in *N. lugens*^4^ (Table 2). In *C. suppressalis*, a BLAST search of our transcriptomic data failed to find NPLP2, NPLP3, and NPLP4.

Proctolin is a pentapeptide with the mature peptide of RYLPT, and it was the first insect neuropeptide to be sequenced and chemically characterized^39^. The first identification of a proctolin precursor gene was CG7105 in *D. melanogaster*^40^. Although a previous study showed that proctolin is absent in *B. mori*^7^, this pentapeptide was recently identified in a proteomic analysis of *B. mori* wings^41^. However, the *Bombyx* proctolin gene does not produce a mature peptide because cleavage sites are not present at the N-terminal and C-terminal of the RYLPT sequence, and a similar gene is observed in *C. suppressalis* (Supplementary Fig. S10). Therefore, a true proctolin has been considered to be not observed in *B. mori* and *C. suppressalis.*

### G protein-coupled receptors (GPCRs) for neuropeptides

**Figure 2:**
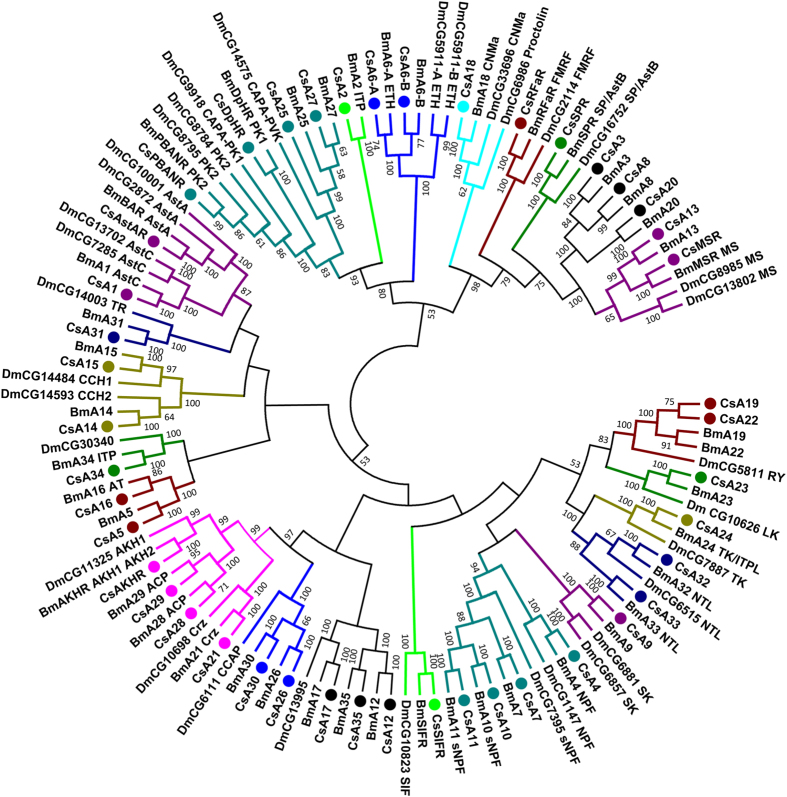
Phylogenetic tree of the A-family neuropeptide GPCRs from *C. suppressalis* (Cs), *B. mori* (Bm), and *D. melanogaster* (Dm). Neighbor-joining trees were constructed using MEGA 5 software with 1000-fold bootstrap re-sampling. The numbers at the nodes of the branches represent the level of bootstrap support for each branch.

**Figure 3:**
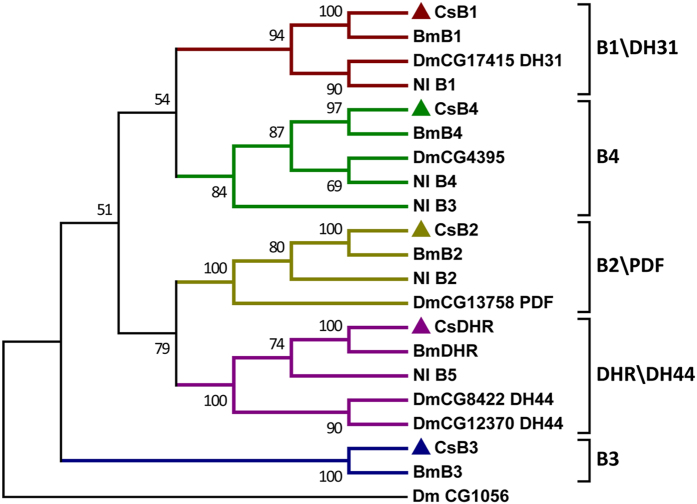
Phylogenetic tree of the B-family neuropeptide GPCRs from *C. suppressalis* (Cs), *B. mori* (Bm), *N. lugens* (Nl), and *D. melanogaster* (Dm). Neighbor-joining trees were constructed using MEGA 5 software with 1000-fold bootstrap re-sampling. The numbers at the nodes of the branches represent the level of bootstrap support for each branch.

**Figure 4:**
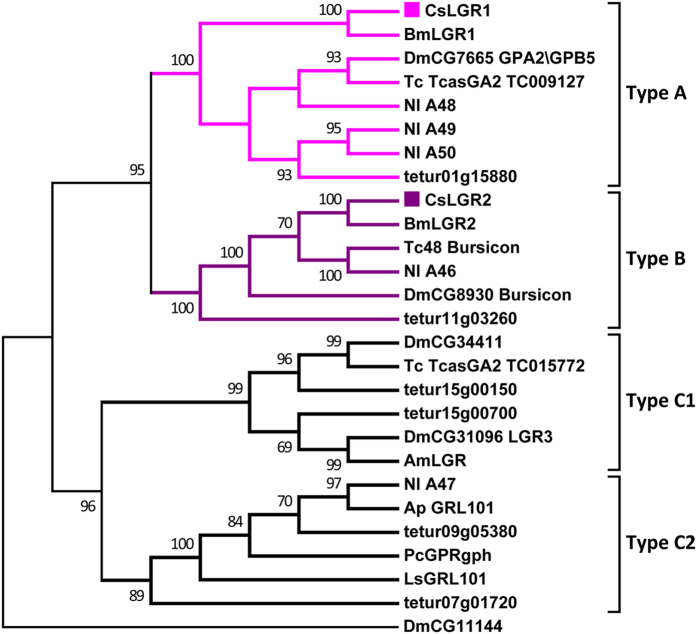
Phylogenetic tree of the leucine-rich repeat-containing GPCRs from *C. suppressalis* (Cs), *B. mori* (Bm), *N. lugens* (Nl), *D. melanogaster* (Dm), *T. castaneum* (Tc), *A. mellifera* (Am), *A. pisum* (Ap), *Pediculus humanus corporis* (Pc), *Lymnaea stagnalis* (Ls), and *Tetranychus urticae* (tetur). Neighbor-joining trees were constructed using MEGA 5 software with 1000-fold bootstrap re-sampling. The numbers at the nodes of the branches represent the level of bootstrap support for each branch.

A total of 51 putative neuropeptide GPCRs were identified in *C. suppressalis* by using all of the homologs of previously categorized *B. mori* and *D. melanogaster* neuropeptide GPCRs as queries (Table 3 and Supplementary Table S1). Based on the predicted amino acid sequences, phylogenetic trees were constructed for the neuropeptide GPCRs of *Chilo*, *Bombyx*, *Drosophila*, and other insects. Of these GPCRs, 44 belonged to the A-family (Fig. 2), 5 belonged to the B-family (Fig. 3), and 2 belonged to the LGRs (Fig. 4). However, the receptors for eclosion hormone (EH), insulin-like peptides (ILPs), neuroparsin (NP), and prothoracicotropic hormone (PTTH) are not GPCRs^42^,^43^,^44^,^45^. In addition, the receptors for *Apis*-ITG-like (ITG), NPLPs, and OKs have not been currently identified in insects.

### A-family GPCRs

In *C. suppressalis*, 44 A-family neuropeptide GPCRs were identified (Fig. 2). Because *C. suppressalis* and *B. mori* are both lepidopteran insects, they have the same number of A-family neuropeptide GPCRs, including neuropeptide receptor A1-A35, adipokinetic hormone receptor (AKHR), allatostatin A receptor (AstAR), diapause hormone receptor (DpHR), FMRFamide receptor (RFaR), myosuppressin receptor (MSR), pheromone biosynthesis activating neuropeptide receptor (PBANR), sex peptide receptor (SPR), and SIFamide receptor (SIFR) (Fig. 2).

In *B. mori*, neuropeptide receptor A2 and A34 have been recently deorphanized as ITP receptors, whereas A24 acts as an ITPL receptor^5^. Additionally, A24 has also been identified as a receptor for tachykinin (TK)^46^. A previous study demonstrated that the TKs and ITPL in *B. mori* have a stimulatory effect on feeding, it is possible that ITPL and TK signaling during the regulation of feeding behavior may directly affect one another by interacting with the receptor, neuropeptide receptor A24^5^.

In the phylogenetic tree, the high-confidence clade shows the relationships between the various GPCRs for RY, LKs, TKs, and NTL, including the neuropeptide receptor A19, A22, A23, A24, A32, and A33 (Fig. 2). NTL is the most recently identified neuropeptide, and its receptors have been deorphanized. In *B. mori*, A32 and A33 have been deorphanized as NTLRs and distinguished because A32 is specific to the FXXXRa motif of NTL and A33 is specific to the YXXXRa motif^19^. The two different motifs are conserved in the NTL precursor in lepidopteran species, and the presence of two receptors that differentiate the two ligand motifs suggests that the two signaling systems diverged at an early evolutionary stage in Lepidoptera^19^.

The clade of the NPF and sNPF receptors is shown in the phylogenetic tree (Fig. 2). Neuropeptide receptor A4 has been identified as a NPF receptor^47^, whereas A10 and A11 are sNPF receptors^14^. Although A7 clusters nicely with A10 and A11, the ligand binding specificity of A7 has not been determined.

Our phylogenetic analysis identified the GPCR clade for the receptors of the GnRH-related peptides and CCAP, including the neuropeptide receptor A21, A26, A28, A29, A30, and AKHR (Fig. 2). A21 acts as a corazonin receptor^48^, whereas A26 and A30 are located near the previously characterized *Drosophila* CCAP receptor CG6111. In *B. mori*, AKHR is activated by AKH1 and AKH2 with a high affinity and by ACP with a low affinity, whereas A28 and A29 are activated by ACP at a high affinity and by AKH1 and AKH2 at a low affinity^49^.

A subtree containing the PK and PVK receptors is shown in the phylogenetic tree (Fig. 2). Neuropeptide receptor A25 and A27 are orthologous to the *Drosophila* CAPA-PVK receptor CG14575. DpHR is fairly exclusive to the *Drosophila* CAPA-PK1 receptor CG9918, whereas PBANR clusters with the *Drosophila* PK2 receptor CG8784 and CG8795.

In the phylogenetic tree, the high-confidence clade contains the GPCRs for CNMamide, FMRFamide, myosuppressin (MS), and allatostatin B (AstB) as well as a number of orphan GPCRs (Fig. 2). Neuropeptide receptor A18 has been identified as a CNMamide receptor (CNMaR)^37^, which is placed near *Drosophila* CNMaR CG33696 and *Drosophila* proctolin receptor CG6986. MSR and A13 cluster nicely with *Drosophila* MSR CG8985 and CG13802. Unfortunately, the neuropeptide receptor A3, A8, and A20 have not been deorphanized.

Additionally, the neuropeptide receptor A12, A17, and A35 cluster with *Drosophila* CG13995 (Fig. 2), and they are all orphan neuropeptide GPCRs.

### B-family GPCRs

Five putative B-family GPCRs were identified in *C. suppressalis* (Fig. 3). Neuropeptide receptor B1 and B2 are orthologous to *Drosophila* CG17415 and CG13758, which have been annotated as diuretic hormone 31 (DH31) receptor and pigment-dispersing factor (PDF) receptor, respectively^50^. These receptors are both calcitonin-like receptors that are involved in the regulation of Ca^2+^ homeostasis. Diuretic hormone-like receptor was also identified in this family, and it is close to the *Drosophila* diuretic hormone 44 (DH44) receptor CG8422 and CG12370, which are corticotropin releasing factor-related receptors. Additionally, the neuropeptide receptor B4 that is homologous to *Drosophila* CG4395 and B3 have not been deorphanized.

### Leucine-rich repeat-containing GPCRs (LGRs)

Three distinct types of LGRs (type A, B, and C) have been defined based on their structural characteristics, and they are distinguished by the number of leucine-rich repeat (LRR) motifs, the absence or presence of an LDLa motif (low density lipoprotein receptor domain class A) and their type-specific hinge region^51^. Two putative LGRs (LGR1 and LGR2) were identified in *C. suppressalis* (Fig. 4). LGR1 clusters with *Drosophila* CG7665 (DLGR1), which is an insect receptor for glycoprotein hormones. LGR1 contains eight LRRs, which are a characteristic feature of type A LGRs. Glycoprotein hormones are heterodimers consisting of α-subunit (GPA2) and β-subunit (GPB5) assembled by noncovalent bonds^52^. In *D. melanogaster*, GPA2 and GPB5 function via DLGR1^52^, which is located in the epithelial cells of the hindgut where it increases the production of cAMP when stimulated by GPA2/GPB5. Hindgut cAMP stimulates the reabsorption of water^53^, and DLGR1 has been reported to play a critical role in development because its role in the regulation of pupariation^54^. LGR2 clusters with Tc48 and *Drosophila* CG8930 (DLGR2), which are receptors for bursicon. LGR2 belongs to type B LGRs characterized by the presence of 16–18 LRRs, which is roughly twice the number of LRRs found in the other types of LGRs. In *D. melanogaster*, bursicon mediates the tanning process in newly emerged adults via DLGR2, which is encoded by the rickets gene. Once activated, DLGR2, elicits the cAMP/PKA signaling pathway, which activates tyrosine hydroxylase, a key enzyme responsible for tanning agent synthesis^55^.

### Expression profiles of neuropeptide precursors and neuropeptide GPCRs

**Figure 5:**
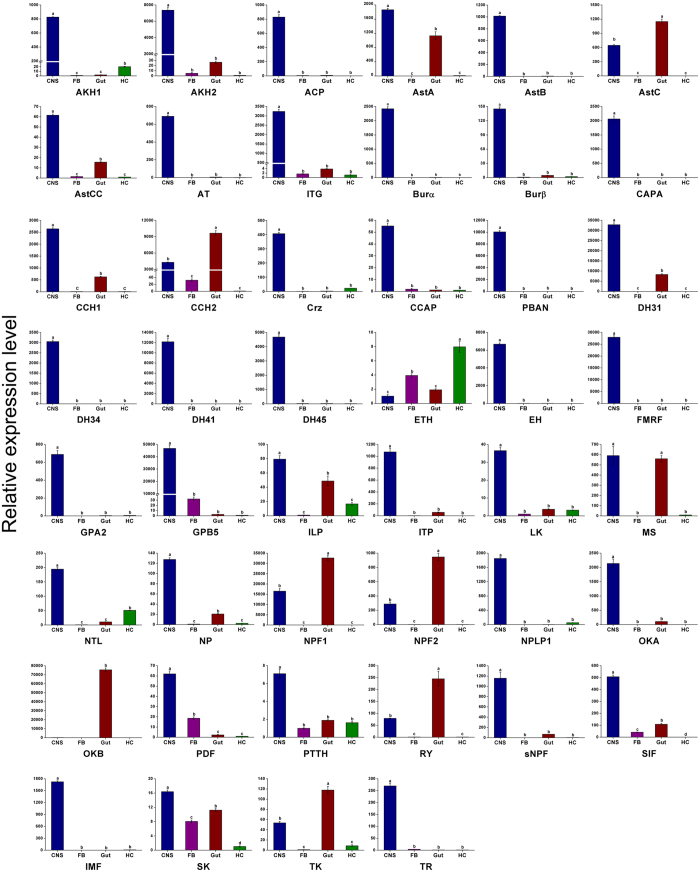
qRT-PCR results showing the relative expression levels of the neuropeptides in various tissues of *C. suppressalis*. Standard errors are represented by the error bars, and significant differences are represented by the different letters above each bar (*p* < *0.05*).

The tissue-specific expression profiles of the neuropeptide precursors and neuropeptide GPCRs in *C. suppressalis* were determined by qRT-PCR from various tissues, including the CNS (brain, suboesophageal ganglion, thoracic ganglion, and abdominal ganglion), the gut (foregut, midgut, hindgut, and Malpighian tubes), hemocytes (HC), and fat body (FB). Of the expression profiles for the neuropeptide precursors, only ecdysis triggering hormone (ETH) was most strongly expressed in the HC, whereas AstC, CCH2, NPF1, NPF2, RY, and TK were predominately expressed in the gut. Interestingly, OKB was expressed only in the gut. Most of the other neuropeptide precursors were preferentially expressed in the CNS (Fig. 5). AstC, NPF, OK, and TK are conserved brain-gut peptides, and their functions include myotropic effects and regulation of feeding behavior^56^. Our findings indicate that they may play a significant role in the digestive system.

**Figure 6:**
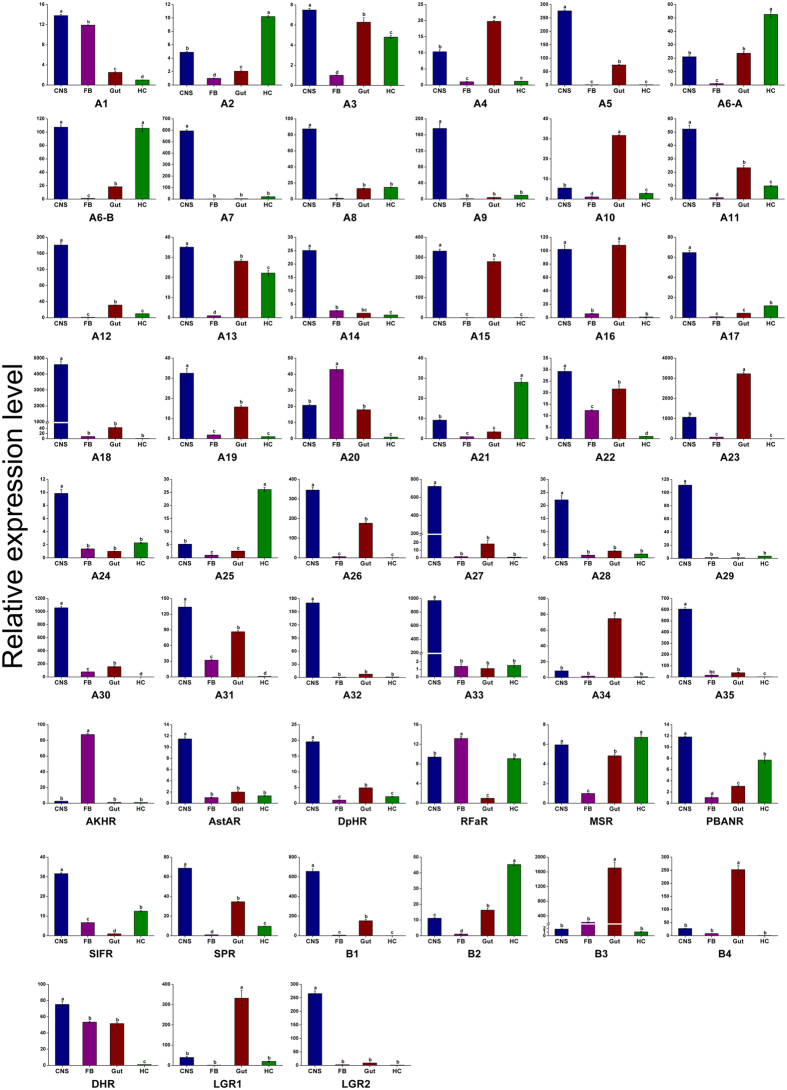
qRT-PCR results showing the relative expression levels of G protein-coupled receptors for the neuropeptides in various tissues of *C. suppressalis*. Standard errors are represented by the error bars, and significant differences are represented by the different letters above each bar (*p* < *0.05*). Neuropeptide receptor A1-A35 are abbreviated as A1-A35, respectively; Neuropeptide receptor B1-B4 are abbreviated as B1-B4, respectively.

The neuropeptide GPCRs, A20, AKHR and RFaR had the highest expression in the FB, whereas A2, A6-A, A6-B, A21, A25, MSR, and B2 were predominately expressed in the HC and A4, A10, A16, A23, A34, B3, B4, and LGR1 had the highest expression in the gut. Additionally, most of the other neuropeptide GPCRs were predominately expressed in the CNS (Fig. 6). Our results showed that most of neuropeptide precursors and their GPCR genes were limited to the CNS, and numerous previous studies have indicated that neuropeptides and their GPCRs play crucial roles in neuromodulation and many other physiological processes in insects.

## Conclusions

RNA-seq is a useful tool for defining the transcriptome of an organism, even when a reference genome is not available. Our transcriptomic analysis of neuropeptide precursors and their putative GPCRs revealed the neuropeptidergic signaling systems in *C. suppressalis*. Our results elucidated the specific characteristics and expression profiles of the neuropeptide precursors and their putative GPCRs in *C. suppressalis*, and provided fundamental information to enhance our understanding of neuro-hormone mechanisms. The data from this study provide the first comprehensive description of neuropeptides and neuropeptide GPCRs in lepidopteran pests. We believe that this data will contribute to pharmacological research into the design of peptidomimetics, pseudopeptides or small molecules capable of disrupting physiological processes regulated by signaling molecules and their GPCRs and will provide beneficial insights into insect pest control.

## Methods

### Insect rearing

The *C. suppressalis* colony has been continuously reared in our laboratory. Larvae were originally collected from a rice field in Fuyang, Zhejiang Province, China, in 2012. The larvae were reared on an artificial diet^57^ and maintained at a temperature of 25 ± 1 °C, relative humidity of 80%, and a light:dark cycle of 14:10.

### RNA-seq

To identify the genes encoding neuropeptides and their putative GPCRs in *C. suppressalis*, RNA samples of the fifth instar larval CNS were prepared for RNA-seq, because larvae are the targets for control and most neuropeptide-related genes are predominately expressed in the CNS. CNS samples (the brain, the suboesophageal ganglion, the thoracic ganglion, and the abdominal ganglion) of 100 fifth instar larvae were individually dissected in a saline solution containing an RNase inhibitor (TaKaRa, Japan). Transcriptome sequencing was performed on an Illumina HiSeq 2000 platform (Novogene Bioinformatics Technology Co.Ltd, Beijing, China), and resulted in 142,051,094 bp raw reads, and these raw reads were then subjected to *de novo* assembly using Trinity software^13^. The transcriptomic data were submitted to the Sequence Read Archive (SRA) database under the accession number SRX1022691^15^.

### Identification of the neuropeptides and their putative G protein-coupled receptors

We used the amino acid sequences of the neuropeptides and GPCRs of the silkworm *B. mori*, the fruit fly *D. melanogaster* and other insects as BLAST queries to search for the candidate sequences of neuropeptides and GPCRs from our *C. suppressalis* transcriptomic data. The BLAST + 2.2.23 software (downloadable from the National Center for Biotechnology Information, Bethesda, MD, USA; ftp://ftp.ncbi.nlm.nih.gov/blast/executables/blast+/) was used for local BLAST search of the assembled unigenes. After gene identification, we used the BLASTX and BLASTN programs against the non-redundant protein (Nr) and non-redundant nucleotide (Nt) NCBI database to identify homologous sequences in other insects.

### Structure and domain analyses and sequence alignments

To identify neuropeptide signal peptide, we used SignalP 4.0 58 (http://www.cbs.dtu.dk/services/SignalP/). The predicted transmembrane domains of the putative neuropeptide GPCRs were verified using TMHMM (http://www.cbs.dtu.dk/services/TMHMM/). For the domain analysis, we used the NCBI Conserved Domain (http://www.ncbi.nlm.nih.gov/Structure/cdd/wrpsb.cgi). Multiple alignments of the amino acid sequences were performed with ClustalX2 59 and edited with GeneDoc software.

### Phylogenetic analysis

We compared the neuropeptide GPCRs of *C. suppressalis* with the neuropeptide GPCRs from *D. melanogaster*, *B. mori* and other arthropods. For the *Drosophila* sequences, the CG numbers were used, whereas for the other species, the originally published names of the GPCRs were used, and the names of identified ligands were added^4^. The phylogenetic trees were constructed with MEGA5.0 60 using the neighbor-joining method. The reliability of each tree node was evaluated by bootstrap proportions using 1000 times.

### Tissue-specific expression analysis

To study the tissue-specific expression profiles of the neuropeptides and their putative GPCRs, total RNA was extracted from various tissues, including the CNS, gut (including the foregut, midgut, hindgut, and Malpighian tubes), hemocytes (HC) and fat body (FB) of fifth instar larvae using TRIzol reagent (Invitrogen, Carlsbad, CA, USA) following the manufacturer’s instructions. For the hemocyte collection, the fifth instar larvae were surface-sterilized with 75% ethanol and the total hemolymph was collected with a 20 μl sterilized pipette by cutting its proleg, and then centrifuged at 200 × g for 10 min at 4 °C to collect the hemocyte precipitate. Other tissues were dissected from the fifth instar larvae on ice. The TransScript One-Step gDNA Removal and cDNA Synthesis SuperMix kits (Transgen, Beijing, China) were used to synthesize cDNA from 1 μg RNA. Specific primers for the qRT-PCR analysis were designed with Primer 3 (http://bioinfo.ut.ee/primer3-0.4.0/) (Supplementary Table S2 and Table S3). The CFX Connect™ Real-Time Detection System (Bio-rad, USA) was used to conduct the qRT-PCR analysis. The reference gene elongation factor 1 alpha (EF-1) was used to normalize the expression of the target genes. The qRT-PCR procedure was performed in a 25 μl reaction containing 12.5 μl SYBR^®^ Premix Ex Taq™ II (Tli RNaseH Plus) (TaKaRa, Japan), 1 μl of each primer (10 μM), 5 μl of cDNA template, and 5.5 μl of sterile H_2_O. The conditions for the qRT-PCR procedure were as follows: 95 °C for 30 s, and then 40 cycles of 95 °C for 5 s and 60 °C for 30 s. The PCR products were then heated to 95 °C for 15 s, cooled to 60 °C for 1 min and heated to 95 °C for 30 s and cooled to 60 °C for 15 s to measure the dissociation curves. Three biological replicates of each tissue were used to ensure the reliability and reproducibility of the results.

The relative quantification of each tissue was calculated using the comparative 2^−ΔΔCT^ method^61^. All of the data were normalized to the endogenous EF-1 levels from the same individual samples. In the analysis of the relative expression level in different tissues, the lowest expression level was used as the calibrator. Thus, the relative expression level in different tissues was assessed by comparing the expression level of each target gene in other tissues with that in the tissue with the lowest expression. The results were presented as the mean of the expression level of three biological replicates. The relative expression levels in the various tissues were analyzed using a one-way analysis of variance (ANOVA) followed by Tukey’s honestly significant difference (HSD) test to determine whether significant differences occurred. All of the statistical analyses were performed by the Data Processing System (DPS) software package (Version 9.5)^62^.

## Additional Information

**How to cite this article**: Xu, G. *et al.* Identification and expression profiles of neuropeptides and their G protein-coupled receptors in the rice stem borer *Chilo suppressalis. Sci. Rep.* **6**, 28976; doi: 10.1038/srep28976 (2016).

## Supplementary Material

Supplementary Information

## Figures and Tables

**Table 1:** Neuropeptides identified from *C. suppressalis.*

Gene name	Accession No.	Acronym	ORF(aa)	SP(aa)	Homology search with known protein		
					Species	E-value	Protein ID
Adipokinetic hormone 1	KT005945	AKH1	67	20	*Bombyx mori*	5e-29	NP_001104825.1
Adipokinetic hormone 2	KT005946	AKH2	73	20	*Bombyx mori*	3e-20	NP_001124365.1
AKH/corazonin-related peptide	KT005947	ACP	86	23	*Helicoverpa armigera*	8e-30	AGH25546.1
Allatostatin A	KT005948	AstA	220	18	*Helicoverpa armigera*	4e-102	O44314.1
Allatostatin B	KT005949	AstB	283	23	*Helicoverpa armigera*	9e-106	AGH25567.1
Allatostatin C	KT005950	AstC	125	27	*Mythimna unipuncta*	8e-54	AAA93257.1
Allatostatin CC splicing variant a	KT005951	AstCCa	140	18	*Bombyx mori*	7e-25	XP_004932108.1
Allatostatin CC splicing variant b	KT005952	AstCCb	106	18	*Bombyx mori*	6e-30	XP_004932108.1
Allatotropin	KT005953	AT	131	20	*Manduca sexta*	5e-72	AAB08757.1
*Apis*-ITG-like	KT005954	ITG	220	21	*Helicoverpa armigera*	3e-134	AGH25548.1
Bursicon alpha subunit	KT005955	Burα	156	25	*Manduca sexta*	1e-81	Q4FCM6.1
Bursicon beta subunit	KT005956	Burβ	137	24	*Manduca sexta*	1e-68	ABB92831.1
CAPA splicing variant a	KT005957	CAPAa	155	19	*Manduca sexta*	2e-60	AAT69684.1
CCHamide 1 splicing variant a	KT005958	CCH1a	187	42	*Bombyx mori*	5e-40	XP_004930537.1
CCHamide 1 splicing variant b	KT005959	CCH1b	162	45	*Bombyx mori*	1e-45	XP_004930537.1
CCHamide 2	KT005960	CCH2	133	22	*Helicoverpa armigera*	4e-61	AGH25550.1
Corazonin	KT005961	Crz	105	19	*Bombyx mori*	4e-37	NP_001036899.1
Crustacean cardioactive peptide	KT005962	CCAP	128	23	*Helicoverpa armigera*	2e-62	AGH25552.1
Diapause hormone/phermone biosynthesis activating neruopeptide	KT005963	PBAN	196	22	*Omphisa fuscidentalis*	1e-53	AFP87384.1
Diuretic hormone 31	KT005964	DH31	111	24	*Helicoverpa armigera*	2e-58	AGH25553.1
Diuretic hormone 41/corticotropin-releasing factor (CRF-DH)	KT005965	DH41	139	17	*Helicoverpa armigera*	7e-62	AGH25554.1
Diuretic hormone 34/splicing variant of CRF-DH	KT005966	DH34	132	17	*Helicoverpa armigera*	2e-45	AGH25555.1
Diuretic hormone 45/splicing variant of CRF-DH	KT005967	DH45	148	17	*Bombyx mori*	1e-53	NP_001124368.1
Eclosion hormone	KT005968	EH	89	26	*Helicoverpa armigera*	3e-39	AAV69026.1
Ecdysis triggering hormone	KT005969	ETH	107^a^		*Danaus plexippus*	3e-29	EHJ75233.1
FMRFamide	KT005970	FMRF	188	22	*Helicoverpa armigera*	4e-83	AGH25556.1
Glycoprotein hormone alpha 2	KT005971	GPA2	115	17	*Helicoverpa armigera*	2e-67	AGH25557.1
Glycoprotein hormone beta 5	KT005972	GPB5	152	No^b^	*Bombyx mori*	7e-69	NP_001124380.1
IMFamide	KT005973	IMF	76	28	*Helicoverpa armigera*	7e-32	AGH25559.1
Insulin-like peptide	KT005974	ILP	127	20	*Bombyx mori*	6e-11	NP_001233285.1
Ion transport peptide-like	KT005975	ITPL	116	22	*Manduca sexta*	7e-58	AAY29658.1
Ion transport peptide	KT005976	ITP	112	22	*Manduca sexta*	7e-56	AAY29657.1
Leucokinin	KT005977	LK	347	18	*Helicoverpa armigera*	2e-154	AGH25561.1
Myosuppressin	KT005978	MS	97	25	*Helicoverpa armigera*	6e-45	AGH25562.1
Natalisin	KT005979	NTL	490	22	*Danaus plexippus*	3e-85	EHJ74348.1
Neuroparsin	KT005980	NP	87	22	*Bombyx mori*	1e-38	NP_001124362.1
Neuropeptide F 1 splicing variant a	KT005981	NPF1a	83	22	*Manduca sexta*	7e-38	AGH20044.1
Neuropeptide F 1 splicing variant b	KT005982	NPF1b	123	22	*Manduca sexta*	1e-60	AGH20043.1
Neuropeptide F 2	KT005983	NPF2	93	21	*Helicoverpa armigera*	1e-55	AEE01342.1
Neuropeptide-like precursor 1	KT005984	NPLP1	334	31	*Bombyx mori*	8e-167	NP_001124353.1
Orcokinin A	KT005985	OKA	195	21	*Bombyx mori*	4e-51	NP_001124366.1
Orcokinin B	KT005986	OKB	226	21	*Danaus plexippus*	3e-44	EHJ77769.1
Pigment dispersing factor	KT005987	PDF	108	22	*Bombyx mori*	6e-22	NP_001036920.2
Prothoracicotropic hormone	KT005989	PTTH	223	28	*Spodoptera exigua*	4e-74	AAT64423.2
Short neuropeptide F splicing variant a	KT005990	sNPFa	219	24	*Bombyx mori*	3e-76	NP_001127729.1
Short neuropeptide F splicing variant b	KT005991	sNPFb	175	24	*Bombyx mori*	3e-94	NP_001127729.1
SIFamide	KT005992	SIF	69	22	*Helicoverpa armigera*	2e-27	AGH25569.1
Sulfakinin	KT005993	SK	74	24	*Helicoverpa armigera*	3e-18	AGH25570.1
Tachykinin	KT005994	TK	255	21	*Helicoverpa armigera*	8e-99	AGH25571.1
RYamide	KT005995	RY	154	34	*Bombyx mori*	1e-15	XP_004925011.1
Trissin	KT005996	TR	114	21	*Bombyx mori*	3e-38	XP_004926342.1

ORF, open reading frame; SP, signal peptide;

^a^not full length; ^b^no signal peptide.

**Table 2:** Neuropeptide genes in *C. suppressalis* and other insects.

Peptide name(acronyms)	*C. suppressalis*	*B. mori*	*D. melanogaster*	*N. lugens*	*A. mellifera*	*T. castaneum*
Adipokinetic hormone 1 (AKH1)	+	+	+	+	+	+
Adipokinetic hormone 2 (AKH2)	+	+	nd	+	nd	+
AKH/corazonin-related peptide (ACP)	+	+	nd	nd	nd	+
Allatostatin A (AstA)	+	+	+	+	+	nd
Allatostatin B (AstB, MIP, PTSP)	+	+	+	+	nd	+
Allatostatin C (AstC)	+	+	+	+	+	+
Allatostatin CC splicing variant a (AstCCa)	+	+	+	+	+	+
Allatostatin CC splicing variant b (AstCCb)	+					
Allatotropin (AT)	+	+	nd	+	+	+
Apis-ITG-like (ITG)	+	+	+	+	+	+
Arginine–vasopressin-like peptide (AVLP)	nd	nd	nd	nd	nd	+
Bursicon alpha subunit (Burα)	+	+	+	+	+	+
Bursicon beta subunit (Burβ)	+	+	+	+	+	+
CAPA splicing variant a (CAPAa)	+	+	+	+	+	+
CCHamide 1 splicing variant a (CCH1a)	+	+	+	+	+	+
CCHamide 1 splicing variant b (CCH1b)	+					
CCHamide 2 (CCH2)	+	+	+	+	+	+
CNMamide	nd	nd	+	+	+	+
Corazonin (Crz)	+	+	+	+	+	nd
Crustacean cardioactive peptide (CCAP)	+	+	+	+	+	+
Diapause hormone/PBAN/Pyrokinin 2	+	+	+	+	+	+
Diuretic hormone 31 (DH31)/Calcitonin-like peptide	+	+	+	+	+	+
Diuretic hormone 41 (DH41)/Corticotropin releasing factor (CRF-DH)	+	+	+	+	+	+
Diuretic hormone 34 (DH34)/splicing variant of CRF-DH	+	+	nd	nd	nd	+
Diuretic hormone 45 (DH45)/splicing variant of CRF-DH	+	+	nd	nd	nd	nd
Ecdysis triggering hormone (ETH)	+	+	+	+	+	+
Eclosion hormone (EH)	+	+	+	+	+	+
FMRFamide (FMRF)	+	+	+	+	+	+
Glycoprotein hormone alpha 2 (GPA2)	+	+	+	+	nd	+
Glycoprotein hormone beta 5 (GPB5)	+	+	+	+	nd	+
IMFamide (IMF)	+	+	nd	nd	nd	nd
Insulin-like peptide (ILP)	+	+	+	+	+	+
Ion transport peptide/Crustacean hyperglycemic hormone (ITP)	+	+	+	+	+	+
Ion transport peptide-like (ITPL)	+	+	+	+	+	+
Leucokinin (LK)	+	+	+	+	+	nd
Myosuppressin (MS)	+	+	+	+	+	+
Natalisin (NTL)	+	+	+	+	+	+
Neuroparsin (NP)	+	+	nd	+	+	+
Neuropeptide F 1 splicing variant a (NPF1a)	+	+	+	+	nd	nd
Neuropeptide F 1 splicing variant b (NPF1b)	+	+	+	+	nd	nd
Neuropeptide F 2 (NPF2)	+	+	nd	+	+	nd
Neuropeptide-like precursor 1 (NPLP1)	+	+	+	+	+	+
Neuropeptide-like precursor 2 (NPLP2)	nd	nd	+	nd	+	nd
Neuropeptide-like precursor 3 (NPLP3)	nd	nd	+	+	+	nd
Neuropeptide-like precursor 4 (NPLP4)	nd	nd	+	+	nd	nd
Orcokinin A (OKA)	+	+	+	+	+	+
Orcokinin A (OKB)	+	+	+	+	+	+
Pigment dispersing factor (PDF)	+	+	+	+	+	nd
Proctolin (Pro)	nd	nd	+	+	nd	+
Prothoracicotropic hormone (PTTH)	+	+	+	+	nd	+
RYamide (RY)	+	+	+	+	+	+
Short neuropeptide F splicing variant a (sNPFa)	+	+	+	+	+	+
Short neuropeptide F splicing variant b (sNPFb)	+					
SIFamide (SIF)	+	+	+	+	+	+
Sulfakinin (SK)	+	+	+	+	+	+
Tachykinin (TK)	+	+	+	+	+	+
Trissin (TR)	+	+	+	nd	nd	+

The data of other insects are referred from *B. mori*^7^, *D. melanogaster*^63^, *T. castaneum*^22^ , *N. lugens*^4^, *A. mellifera*^21^.

+, identified; nd, not identified.

**Table 3:** G protein-coupled receptors for neuropeptides identified from *C. suppressalis.*

Gene name	Accession No.	Length	ORF	Putative identification	Species	Accession No.	E-value
Neuropeptide receptor A1	KT030998	2170	1248	Allatostatin receptor	*Manduca sexta*	ADX66345.1	0
Neuropeptide receptor A2	KT030999	1135	1014	Neuropeptide receptor A2	*Bombyx mori*	NP_001127737.1	5e-128
Neuropeptide receptor A3	KT031000	1386	1314	Neuropeptide receptor A3	*Bombyx mori*	NP_001127738.1	0
Neuropeptide receptor A4	KT031001	1888	1146	Neuropeptide receptor A4	*Danaus plexippus*	EHJ70829.1	0
Neuropeptide receptor A5	KT031002	2679	1692	Neuropeptide receptor A5	*Bombyx mori*	NP_001127740.1	0
Neuropeptide receptor A6-A	KT031003	2170	1737	Ecdysis triggering hormone receptor subtype A	*Manduca sexta*	AAX19163.1	0
Neuropeptide receptor A6-B	KT031004	2235	1695	Ecdysis triggering hormone receptor isoform B	*Bombyx mori*	NP_001165737.1	0
Neuropeptide receptor A7	KT031005	1422	1323	Neuropeptide receptor A7	*Bombyx mori*	NP_001127742.1	0
Neuropeptide receptor A8	KT031006	2869	1272	Neuropeptide receptor A8	*Bombyx mori*	NP_001127743.1	0
Neuropeptide receptor A9	KT031007	3592	1374	Neuropeptide receptor A9	*Bombyx mori*	NP_001127744.1	0
Neuropeptide receptor A10	KT031008	2423	1302	Neuropeptide receptor A10	*Bombyx mori*	NP_001127707.1	0
Neuropeptide receptor A11	KT031009	2842	1392	Neuropeptide receptor A11	*Bombyx mori*	NP_001127708.1	0
Neuropeptide receptor A12	KT031010	1431	1332	Neuropeptide receptor A12	*Bombyx mori*	NP_001127709.1	0
Neuropeptide receptor A13	KT031011	2042	1128	Neuropeptide receptor A13	*Bombyx mori*	NP_001127710.1	0
Neuropeptide receptor A14	KT031012	1879	1230	Neuropeptide receptor A14	*Bombyx mori*	NP_001127711.1	0
Neuropeptide receptor A15	KT031013	1463	1152	Neuropeptide receptor A15	*Bombyx mori*	NP_001127712.1	0
Neuropeptide receptor A16	KT031014	1932	1542	Allatotropin receptor	*Manduca sexta*	ADX66344.1	0
Neuropeptide receptor A17	KT031015	1808	1122	Neuropeptide receptor A17	*Bombyx mori*	NP_001127715.1	9e-169
Neuropeptide receptor A18	KT031016	1952	1302	Neuropeptide receptor A18	*Bombyx mori*	NP_001127716.1	7e-147
Neuropeptide receptor A19	KT031017	1418	1347	Neuropeptide receptor A19	*Bombyx mori*	NP_001127717.1	0
Neuropeptide receptor A20	KT031018	1992	1269	Neuropeptide receptor A20	*Danaus plexippus*	EHJ69284.1	0
Neuropeptide receptor A21	KT031019	1812	1302	Neuropeptide receptor A21	*Bombyx mori*	NP_001127719.1	0
Neuropeptide receptor A22	KT031020	2788	1341	Neuropeptide receptor A22	*Bombyx mori*	NP_001127720.1	0
Neuropeptide receptor A23	KT031021	2134	1449	Neuropeptide receptor A23	*Bombyx mori*	NP_001127721.1	0
Neuropeptide receptor A24	KT031022	1780	1254	Neuropeptide receptor A24	*Bombyx mori*	NP_001127722.1	0
Neuropeptide receptor A25	KT031023	1017		Neuropeptide receptor A25	*Bombyx mori*	NP_001127723.1	2e-105
Neuropeptide receptor A26	KT031024	1712	1314	Neuropeptide receptor A26	*Bombyx mori*	NP_001127724.1	0
Neuropeptide receptor A27	KT031025	1644	1473	Neuropeptide receptor A27	*Bombyx mori*	NP_001127725.1	0
Neuropeptide receptor A28	KT031026	1822	1329	Neuropeptide receptor A28	*Bombyx mori*	NP_001127726.1	9e-171
Neuropeptide receptor A29	KT031027	1190		Neuropeptide receptor A29	*Bombyx mori*	NP_001127745.1	0
Neuropeptide receptor A30	KT031028	1652	1275	Neuropeptide receptor A30	*Bombyx mori*	NP_001127746.1	0
Neuropeptide receptor A31	KT031029	1293		Neuropeptide receptor A31	*Bombyx mori*	NP_001127747.1	0
Neuropeptide receptor A32	KT031030	1169		Neuropeptide receptor A32	*Bombyx mori*	NP_001127748.1	0
Neuropeptide receptor A33	KT031031	2681	1194	Neuropeptide receptor A33	*Bombyx mori*	NP_001127749.1	0
Neuropeptide receptor A34	KT031032	828		Neuropeptide receptor A34	*Bombyx mori*	NP_001127750.1	5e-154
Neuropeptide receptor A35	KT031033	2151	1275	Neuropeptide receptor A35	*Bombyx mori*	NP_001127751.1	0
Adipokinetic hormone receptor	KT031034	2275	1182	Adipokinetic hormone receptor	*Manduca sexta*	ACE00761.1	0
Allatostatin A receptor	KT031035	2260	1083	Allatostatin A receptor	*Spodoptera littoralis*	ACJ06649.1	0
Diapause hormone receptor	KT031036	1914	1353	Diapause hormone receptor	*Ostrinia nubilalis*	AGL12069.1	4e-165
FMRFamide receptor	KT031037	2217	1284	FMRFamide receptor	*Bombyx mori*	NP_001037007.1	0
Myosuppressin receptor	KT031038	1272	1140	Myosuppressin receptor	*Bombyx mori*	NP_001036929.1	0
Pheromone biosynthesis activating neuropeptide receptor-A	KT031039	1672	1041	Pheromone biosynthesis activating neuropeptide receptor-A	*Ostrinia nubilalis*	AGL12066.1	2e-176
Pheromone biosynthesis activating neuropeptide receptor-B	KT031040	1563		Pheromone biosynthesis activating neuropeptide receptor-B	*Ostrinia nubilalis*	AGL12067.1	0
SIFamide receptor	KT031041	1888	1425	SIFamide receptor	*Bombyx mori*	NP_001266380.1	0
Sex peptide receptor	KT031042	2477	1242	Sex peptide receptor	*Spodoptera litura*	AGE92037.1	0
Neuropeptide receptor B1	KT031043	5860	1197	Neuropeptide receptor B1	*Danaus plexippus*	EHJ71642.1	0
Neuropeptide receptor B2	KT031044	951		Neuropeptide receptor B2	*Bombyx mori*	NP_001127733.1	1e-128
Neuropeptide receptor B3	KT031045	2716	2361	Neuropeptide receptor B3	*Bombyx mori*	NP_001127734.1	0
Neuropeptide receptor B4	KT031046	1395	1248	Neuropeptide receptor B4	*Danaus plexippus*	EHJ67831.1	0
Diuretic hormone receptor	KT031047	1345		Diuretic hormone receptor	*Manduca sexta*	P35464.1	0
Leucine-rich repeat G protein-coupled receptor 1	KT031048	2334	2211	Leucine-rich repeat G protein-coupled receptor	*Bombyx mori*	NP_001037033.1	0
Leucine-rich repeat G protein-coupled receptor 2	KT031049	2692		Putative Leucine-rich transmembrane protein	*Danaus plexippus*	EHJ76329.1	0
