# Supplementary material for: Identification and expression profiles of neuropeptides and their G protein-coupled receptors in the rice stem borer Chilo suppressalis
Source: Sci Rep. 2016 Jun 29;6:28976. doi: 10.1038/srep28976 (PMC4926255; doi:10.1038/srep28976)
Supplement: Supplementary Information [file srep28976-s1.pdf]

Supplementary Information:

Identification and expression profiles of neuropeptides and their G protein-coupled receptors in the rice stem borer *Chilo suppressalis*

Gang Xu[1], Gui-Xiang Gu[1], Zi-Wen Teng[1], Shun-Fan Wu[1, 2], Jia Huang[1], Qi-Sheng Song[3], Gong-Yin Ye[1], and Qi Fang[1]*

[1] State Key Laboratory of Rice Biology & Key Laboratory of Agricultural Entomology of Ministry of Agriculture, Institute of Insect Sciences, Zhejiang University, Hangzhou 310058, China

[2] College of Plant Protection, Nanjing Agricultural University, Nanjing 210095, China; State & Local Joint Engineering Research Center of Green Pesticide Invention and Application

[3] Division of Plant Sciences, Missouri University, Columbia, MO 65211, USA

*Corresponding author: Qi Fang, Institute of Insect Sciences, Zhejiang University, Hangzhou 310058, China, Tel: 86-0571-88982696, E-mail: fangqi@zju.edu.cn

This file includes:

Tables S1-S3

Figures S1-S10

Table S1 G protein-coupled receptors with likely ligands in insects.

**Receptor**	**Close family**	**Likely ligands**
Neuropeptide receptor A1	CG7285, CG13702, BmA1	AstC
Neuropeptide receptor A2	BmA2	ITP
Neuropeptide receptor A3	CG13229, BmA3	Orphan
Neuropeptide receptor A4	CG1147, BmA4	NPF
Neuropeptide receptor A5	BmA5	Orphan
Neuropeptide receptor A6-A	CG5911-A, BmA6-A	ETH
Neuropeptide receptor A6-B	CG5911-B, BmA6-B	ETH
Neuropeptide receptor A7	CG7395, BmA7	Orphan
Neuropeptide receptor A8	CG13229, BmA8	Orphan
Neuropeptide receptor A9	CG6881, CG6857, BmA9	SK
Neuropeptide receptor A10	CG7395, BmA10	sNPF
Neuropeptide receptor A11	CG7395, BmA11	sNPF
Neuropeptide receptor A12	BmA12	Orphan
Neuropeptide receptor A13	BmA13	MS
Neuropeptide receptor A14	CG14593, BmA14	CCH2
Neuropeptide receptor A15	CG14484, BmA15	CCH1
Neuropeptide receptor A16	BmA16	AT
Neuropeptide receptor A17	CG13995, BmA17	Orphan
Neuropeptide receptor A18	CG6986, BmA18	CNMa
Neuropeptide receptor A19	CG5811, BmA19	RY
Neuropeptide receptor A20	CG13229, BmA20	Orphan
Neuropeptide receptor A21	CG10698, BmA21	Crz
Neuropeptide receptor A22	CG5811, BmA22	RY
Neuropeptide receptor A23	CG10626, BmA23	LK
Neuropeptide receptor A24	CG7887, BmA24	TK/ITPL
Neuropeptide receptor A25	CG14575, BmA25	CAPA-PVK
Neuropeptide receptor A26	CG6111, BmA26	CCAP
Neuropeptide receptor A27	CG14575, BmA27	CAPA-PVK
Neuropeptide receptor A28	CG11325, BmA28	ACP
Neuropeptide receptor A29	CG11325, BmA29	ACP
Neuropeptide receptor A30	CG6111, BmA30	CCAP
Neuropeptide receptor A31	CG14003, BmA31	TR
Neuropeptide receptor A32	CG6515, BmA32	NTL (FXXXRa)
Neuropeptide receptor A33	CG6515, BmA33	NTL (YXXXRa)
Neuropeptide receptor A34	CG30340, BmA34	ITP
Neuropeptide receptor A35	BmA35	Orphan
Adipokinetic hormone receptor	CG11325, BmAKHR	AKH1, AKH2
Allatostatin A receptor	CG2872, CG10001, BmBAR	AstA
Diapause hormone receptor	CG9918, BmDpHR	CAPA-PK-1
FMRFamide receptor	CG2114, BmRFaR	FMRF
Myosuppressin receptor	CG8985 CG13802 BmMSR	MS
Pheromone biosynthesis activating neuropeptide receptor	CG8784, CG8795 BmPBANR	PK-2
Sex peptide receptor	CG16752, BmSPR	SP/AstB
SIFamide receptor	CG10823, BmSIFR	SIF
Neuropeptide receptor B1	CG17415, BmB1	DH31
Neuropeptide receptor B2	CG13758, BmB2	PDF
Neuropeptide receptor B3	BmB3	Orphan
Neuropeptide receptor B4	CG4395, BmB4	Orphan
Diuretic hormone receptor	CG8422, CG12370, BmDHR	DH41
Leucine-rich repeat G protein-coupled receptor 1	CG8930	GPA2/GPB5
Leucine-rich repeat G protein-coupled receptor 2	CG7665	Bursicon

Table S2 Primers used for the qRT-PCR analysis of neuropeptide genes.

**Primers**	**Forward primer (5'-3')**	**Reverse primer (5'-3')**
AKH1	CGAGTCTCAACTTCAAACAGCA	AGCCGCTATAGCTTCGTCGT
AKH2	GACGCCCAGTTGACCTTCAG	TTTTGCCACCATCTCTACACGA
AKH3	GATCAGGTGTAGGGGCGTGA	TGTCGACAAAGCCTGGTGAA
AstA	CTGGCCCAGTTTACGAAGAG	AAATTGAAACGGTGCAGGTC
AstB	GCGAACTTCCACGGATCTTG	AGGGCTGACCATGCCTTCTT
AstC	CGGGCAACGCTTACTTTATC	ATCCGGACGAACTACAATCG
AstCC	CGCTCCCGTTACTGTTGGTG	GTCGACCTCCATCTGCTCGT
AT	ACCACCAGGGCTGAGCTGTA	ACTGACCGGGTGAAATCAGG
ITG	TACCGCCACTACGCTTACGG	CGCCCTCCGTCATCTGTATC
Burα	CTTCGTCATCCCGGGTGTAA	GGGGCAAAAGAGGACTACCG
Burβ	GCGGGGAAGTCAGTGTCAAC	TCTTCCAGCCTCAAGCCATC
CAPA	AGTCATCGCCAGCGCATATC	CTGACGTTTGGCTGTCATCG
CCH1	GCCAGCTGCGAACAAAGAAC	TCCAACATCGGTTTGGCTTC
CCH2	GGCAAACGATCTGGCGATAC	GCTTCCCTGGCGCTATCTTC
Crz	CGAACCTAAGCCTGCTGCTG	GCCTGCCTTCCAACAAGTTC
CCAP	CAGGTTGTGGTCGCAAGAGA	CTGCGCTTGCATCTTGAATG
DH/PBAN	CGAACAGTTGCCTGCGTATG	TGATCCGGTTGTGTGACTGC
DH31	TGCTCGTCGTACCCACTTCA	CTGAGGCCAAGATCCAATGC
DH41	CAACTACGGTGTGCCACCAA	CCTCTCCTTCTCCAGGCTGA
DH34	CAACTACGGTGTGCCACCAA	CTCTGAAGCACGTCCACAGC
DH45	CAACTACGGTGTGCCACCAA	GCACCTCCATTGGGTTGTTG
EH	TCGTGCTGTTCGCTGTCTTC	ACAGGATTCTGCGCATAGGG
ETH	GCGTTAGTCGCATATTGGCTGT	CTCCCCATTCGTGGGATGT
FMRF	CGCAATCGATCGCAGTATGA	GGGCTGTCGTATTCGTCGTC
GPA2	CCTGGCTGTCATCGAATAGGTC	GTTGCAGCACTGACCCAATG
GPB5	GCAGACGGACCAGAATGGAC	CACTACTCCTGGGTCGCAATG
ILP	CCAGATGCTGGGAGAGATCG	TGACGTAGCAAGCCTTGTGG
ITP	GCATCTGCGACGACTGCTAC	TTCCCGACGAAGTCGATCAT
LK	CATGGGGAGGCAAAAGATCA	GCTTGTCCAGCGAGGGTATG
MS	GAGCACCGAAACCCGTGTAG	GCTCGAGGAAGGTGTTGAGC
NTL	TGAGGGGCAGACGTGATTCT	TTCTTCTTCCCGCCTTCCTC
NP	GTCCCGTGCTGCAAAGAATG	CCAGCCACCGTCTACCCTTA
NPF1	TGAACAAACGCATCGCAGTC	CGGTCTAGCAGCCTGTGTGT
NPF2	GTTGCTGTCCGCCATCTTGT	CATCTGCCTCTTGCCGTACC
NPLP1	AAAATGGCCAACTCCCGACT	CTACCGACGCGGTAACCATC
OKA	AATTTCGTGCGCAAGAGGAA	TGGAACCAATGGGAAACGAC
OKB	ACCGCTGATTCGCTACAGGA	GATGCCAGCGAGGTTTCTTC
PDF	TGCCCAAAGTCTGGCTTGAT	ATCACCATCCGTCCAGTTGC
Pro	CCAAAGCAAGGAACGACAGC	CGCCGCGAACTTTCTTAAAATC
PTTH	ACGGTCATCATGTGCTGTGG	TCCAATGCCATCTTCTGCAC
RY	GGCGTTGATGACGCTAGTGG	TGCCCATGAAGAACCTGTCA
sNPF	TCAGCGTCAGCGGACAGTAG	AGCTCGTCCATTTCGTCCAA
SIF	TGCGTGCAGTATTCGCCATA	ATGCCTGGCAGGTTTCTGAG
IMF	TCGCTGGTGCAAGTAGGAGA	TGGGAACCAAGCTTGACAGG
SK	CTATCTTCGCGTTGGCAGTG	AGATGGCCGTAGTCGTCGAA
TK	TGGGCAAGAGGTGCAGAAAC	GCATGCCTACGAAGCCCATA
TR	CCTGTGGCAGTGAGTGCTTG	TACCACGGTTCAGCCACAGA
EF-1	AAATCGGCGGTATTGGTACG	AAGGGGAGGGAATTCTTGGA

Table S3 Primers used for the qRT-PCR analysis of the neuropeptide receptor genes.

Primers	Forward primer (5'-3')	Reverse primer (5'-3')
A1	GCTTCGCTACTCCAAAATGC	GGTGGCAGACCGCTATGTAT
A2	TTGGCGAAGTAGGATGTCGTC	AATGCCACAGCCCAGATCAC
A3	TTAACCGTCGTGGGATACGTC	CGACGGTTGTGTGAATGTCG
A4	TTCTCGCAGAACAGGAAGGT	GTAGAGGCCAGTGCTTCGTC
A5	GTGGCGGATTTCATGGTGAT	GGAAGCAGATGGCGTACCAG
A6-A	AATGAGAAGCGAAGGGCACA	ATCAAAACGCTTCGCCTCAA
A6-B	ATCGCCAAGAACCTCATGGA	CGCTTTAAATGGCAGCAAGC
A7	GCTGGAACAGCTAGGAGTGG	CCACAGTCAGCAAGTCCTCA
A8	ATGTCGGTTCTACGGGAGCA	AGTAGCCATGGATGCGGTTG
A9	GGGAATGCGCCATGAGATAC	CGGAGTCCAGCTTCTCCTCA
A10	TTTGTTGCGCATTTCATAGC	AACGGTTTCATTACCGTTGC
A11	CGTACGTCCAAGGAACCAGT	TGACCTATCTGAGGGCCAAC
A12	GGATCTTGCTGCTGCTTGCT	TAGCGGAGTTTGCATGACCA
A13	TGGACATGTTGGCGTTGTTC	TTGTGGGCTCCATCTTGGTC
A14	CCTGGTTCCGGTAATGTTCG	CGGCACGCATATCAAGATCA
A15	TGTGTTCGTGAGGCACAAGG	CACTGACACCCCAATGCTGA
A16	GAGAAAAGCTGCCAAAATGC	TGATCGCTGAGTTGGCATAG
A17	CCTCGGCAACGTCCTCATAG	CCAGTATGCCACTGCGTTCA
A18	TCATCGTGTCGTCCGTGTTC	GCGCAAAGTTTATGCCGAAG
A19	TCAGCCAGGGACAACAGGAT	TGACTCGCGAACCACACGTA
A20	TTACGGCACACAACGAATGC	AAGTCTCGCCGAGTCAGCAC
A21	GTGGTCTGCGGGAAACGTAG	GACCAATTTGCCGCTTCTTG
A22	TGATCGTGGTGGCTGTATGG	AGCGCAAACTGGAGAGCAAG
A23	CCACATCGAGACGAATGCAG	GGCAGAAAGGGCACATGAAG
A24	TGCCCATAATGTCCATGTCG	ACCGCGAATATCACCACCAC
A25	TGCTTAGCCTCCGTGCTAGA	ACTACAGCCCCATCCTGACG
A26	ATAGCTGCCCTGCTCTGCAC	ATCACCACAGCCTGGAGGAA
A27	CCGAGCGTTGAGGATAGTGG	CAAGCTGCTCAGCTCGCATA
A28	GTTTGGCGATTGCGAGAAAG	CGCATAGGCAGAACACGTTG
A29	TTGTAGCCTGCCACAGAGCA	TCTCGCAGAAGATGCACACG
A30	CTCGGCAAGAGAGCCACTGT	TACGGCGACCAGCATAGGAC
A31	CTCGTGGTGACGTTGTCGAG	GTAGCTGAGCGAGTGCACGA
A32	ACGTACGCGGTTCCAATGTT	GCCAGCAGATGCCGAATATC
A33	GAAATGGGCGCACCTTAACA	GAGCGTGTTTCCCAAGATGG
A34	TTACTGGAGGGACCGGTTGA	AACAGCACCAAGAGCCGAAC
A35	CACGCTGCTCATCTTCATCG	CCTCGCCGAAGTACCAGATG
AKHR	CGCCTGGATGTACTCCTCAT	GCTATGCAAATCAGCACGAA
AstAR	AGTATCAGCGGAAAGCAGGA	TCTGAAAGCGACCCTGAAGT
DpHR	GGCTTGGCATCAGAAACGTC	GCTTGTGGTAGTGCCAGTGC
RFaR	CGTGTACCTGACGCTCATCG	TCGACGCTCACGTACGTCTC
MSR	ACGGGATTAGCAGTCGCTGA	GGGTGATTGTCAACCAGATCG
PBANR	TTGTGGACATACCGCCAGTG	CGACGCAGGTGCTTATGTTG
SIFR	CTGGATCTTCGCCGTACTCG	CCGTGGGGAGGATGTAACAG
SPR	TCGTGGATCGGCAGTATGTG	ACAGGGGATCAAGTGGACGA
B1	GCCCTGTCCAGACTTTGTGC	CAGCGACACGCTGTAACCTG
B2	TGCCGTATCTTTGCGAACTC	CCCACCCCACAACGTAGTTT
B3	GCAACGCTGTGTTCGTATGC	AGCACGAGTGGTCGTTGTGA
B4	GTCATCGGGTTCGACTCCAG	AAGCAGTGCTGCCACAGACA
DHR	GCGATGAGGTGGAAGAATGC	GGGTGGCATTTTGTGTGTCA
LGR1	GTGTACTCCGAAGCCGAACG	GAGGTGGCACATGAGGAAGC
LGR2	AGGCGCATAACAACCCACAC	AACACGAGGTCGCTGAGTCC
EF-1	AAATCGGCGGTATTGGTACG	AAGGGGAGGGAATTCTTGGA

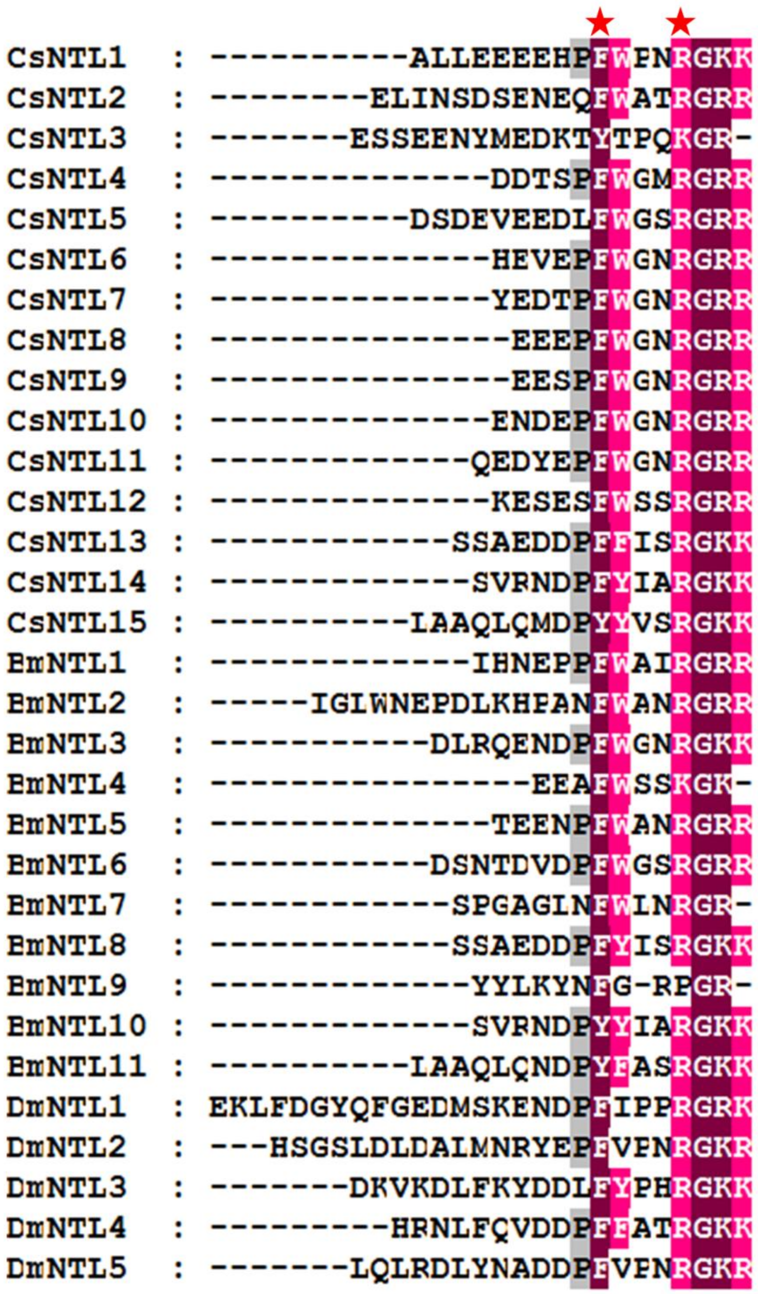

Figure S1 Alignment of natalisins (NTLs) predicted from *C. suppressalis* (Cs), *B. mori* (Bm), and *D. melanogaster* (Dm). Identities are highlighted in red, and similarities are indicated by gray. The red asterisks indicate the characteristics of NTL (FXXXRa, YXXXRa).

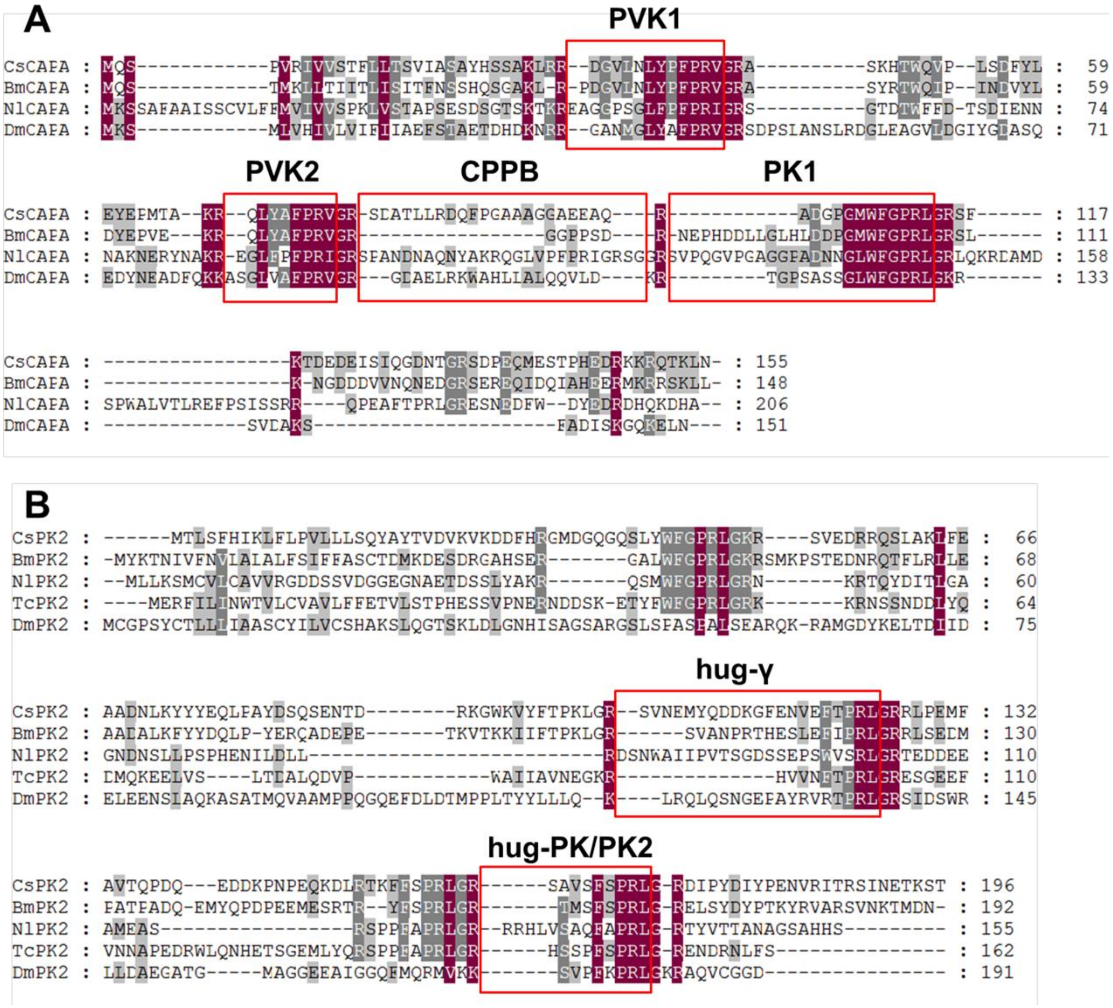

Figure S2 Sequence alignment of CAPA (PK1) **(A)** and DH/PBAN (PK2) **(B)** from *C. suppressalis* (Cs), *B. mori* (Bm), *N. lugens* (Nl), *T. castaneum* (Tc), and *D. melanogaster* (Dm). Identities are highlighted in dark red, and similarities are indicated by gray. The solid boxes indicate the mature peptides. PVK, periviscerokinin; CPPB, CAPA precursor peptide B; PK, pyrokinin.

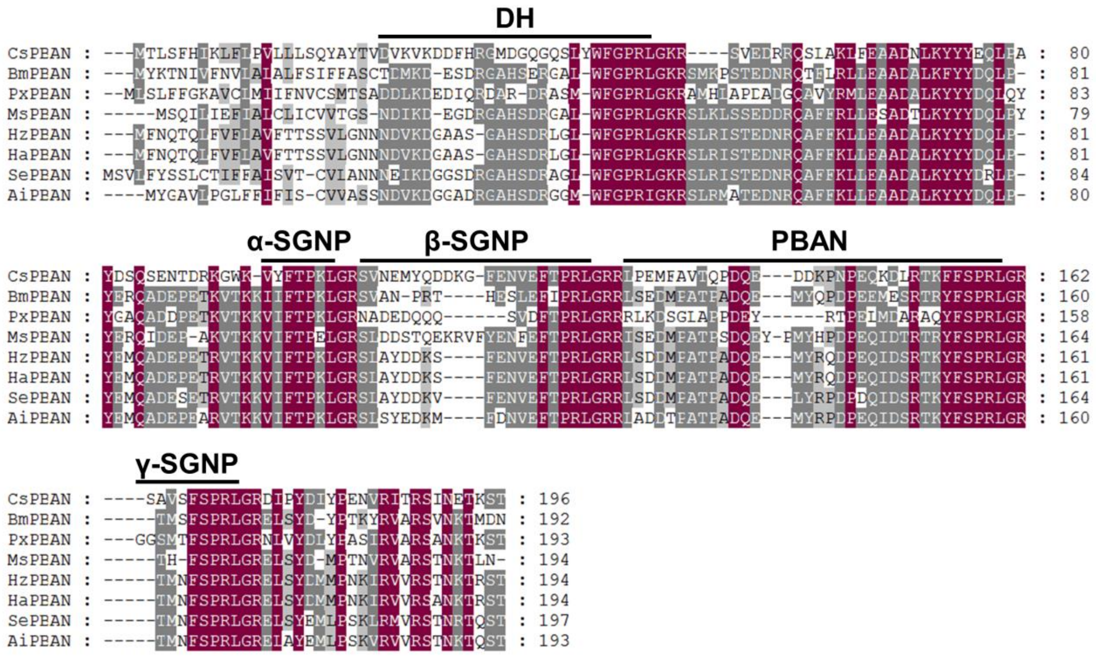

Figure S3 Protein alignment of diapause hormone/PBAN (DH/PBAN) precursor sequences in moths, including *C. suppressalis* (Cs), *B. mori* (Bm), *Plutella xylostella* (Px), *M. sexta* (Ms), *H. zea* (Hz), *H. armigera* (Ha), *S. exigua* (Se), and *Agrotis ipsilon* (Ai). SGNP: subesophageal ganglion neuropeptide. Identities are highlighted in dark red, and similarities are indicated by gray.

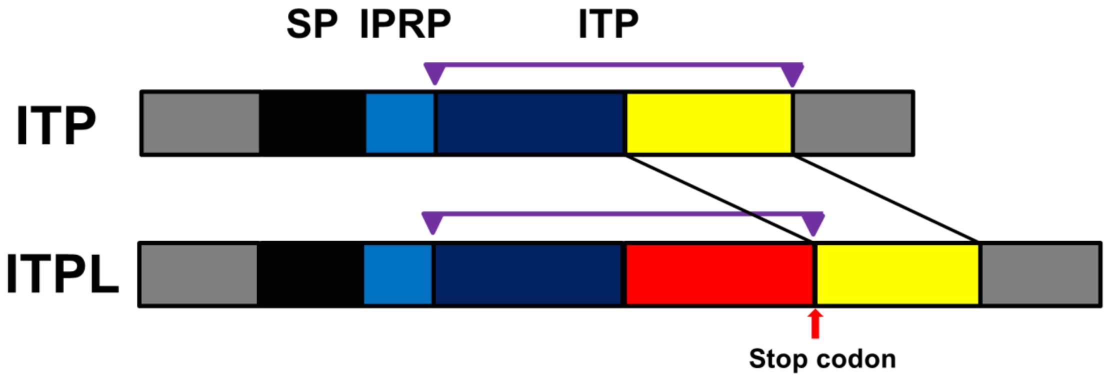

Figure S4 Schematic representation of the highly conserved ITP gene structures that contains exons arranged in tandem and lead to derived alternative mRNA splice forms that encode the common and the distinctive parts of short (yellow, ITP) and long (red, ITPL) peptide isoforms. The red arrow marks the stop codon of ITPL. SP, signal peptide; IPRP, ITP precursor-related peptide.

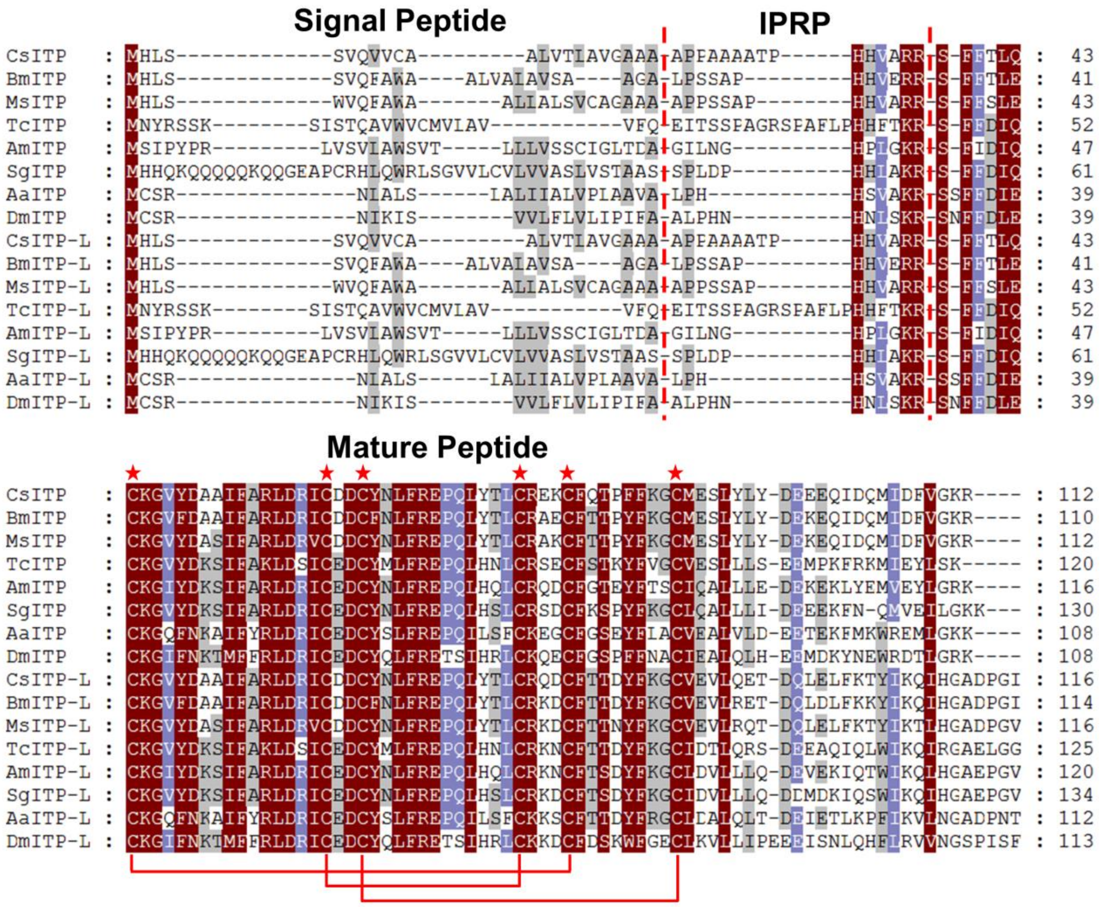

Figure S5 Protein alignment of the ITP and ITPL precursor sequences from *C. suppressalis* (Cs), *B. mori* (Bm), *M. sexta* (Ms), *T. castaneum* (Tc), *A. mellifera* (Am), *S. gregaria* (Sg), *A. aegypti* (Aa), and *D. melanogaster* (Dm). Identities are highlighted in dark red, and similarities are highlighted in light color. The signal peptide, ITP precursor-related peptide (IPRP), and mature peptide are separated by dashed lines. The red asterisks mark the conserved cysteine residues, and three disulfide bridges are indicated.

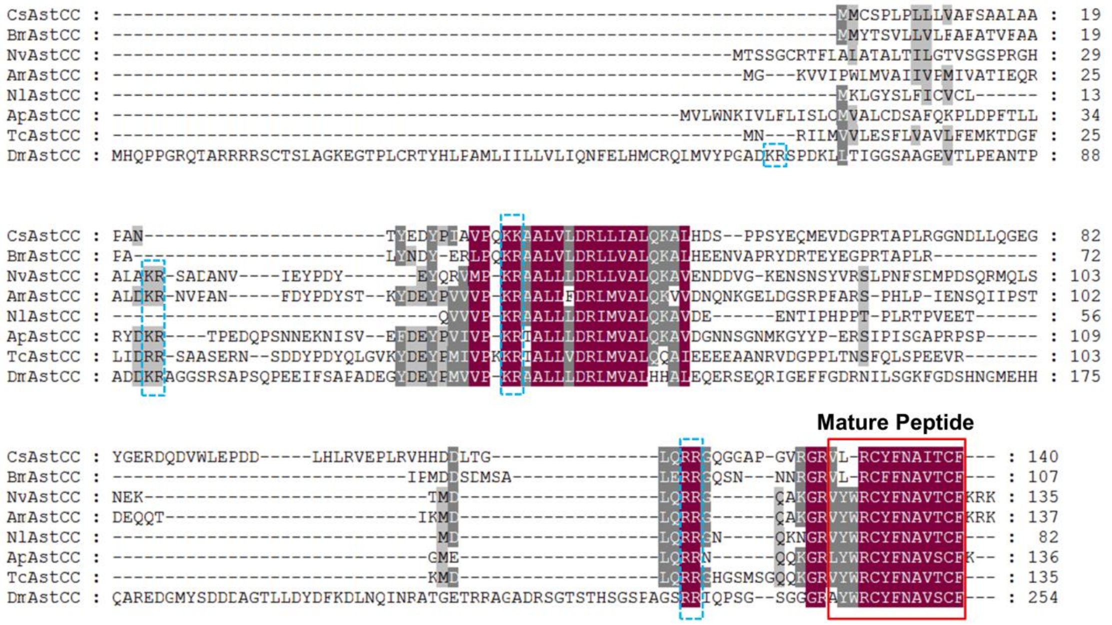

Figure S6 Sequence alignment of the allatostatin double C (AstCC) from *C. suppressalis* (Cs), *B. mori* (Bm), *N. vitripennis* (Nv), *A. mellifera* (Am), *N. lugens* (Nl), *A. pisum* (Ap), *T. castaneum* (Tc), and *D. melanogaster* (Dm). Identities are highlighted in dark red, and similarities are indicated by gray. The solid box indicates the mature peptide, and the dashed boxes indicate Lys and Arg residues which may form convertase cleavages sites or be removed by carboxypeptides.

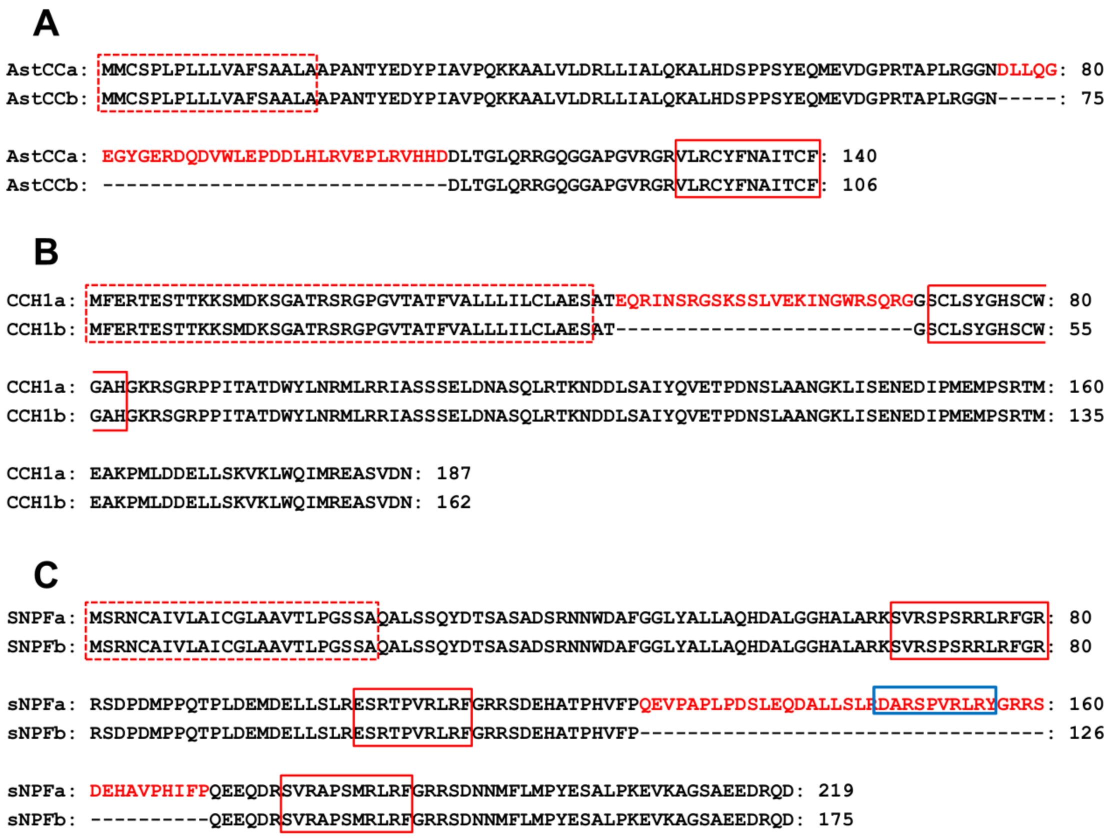

Figure S7 Protein alignment of novel alternative splicing variants of three neuropeptide precursor genes from *C. suppressalis*. (A) AstCC; (B) CCH1; (C) sNPF. The dashed boxes indicate the signal peptides, the solid red boxes indicate the mature peptides, and the solid blue box indicates one more mature peptide of sNPF. The red texts represent the differences between two splicing variants.

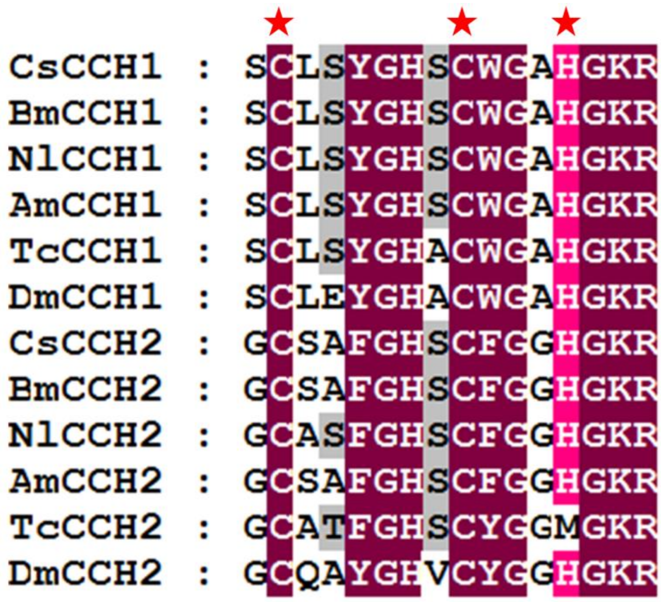

Figure S8 Alignment of CCHamides predicted from *C. suppressalis* (Cs), *B. mori* (Bm), *N. lugens* (Nl), *A. mellifera* (Am), *T. castaneum* (Tc), and *D. melanogaster* (Dm). Identities are highlighted in dark red, and similarities are indicated by gray. The red asterisks mark the conserved residues explaining the etymology of CCHamide.

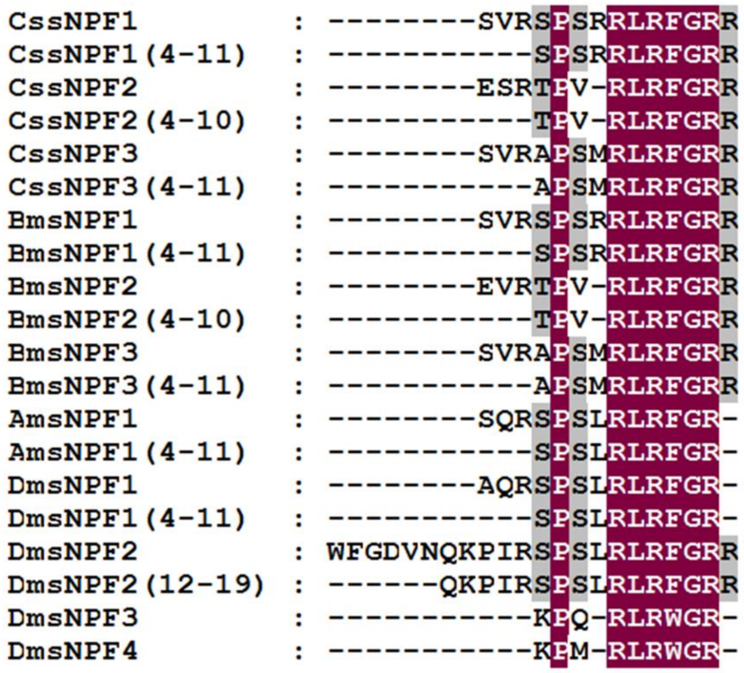

Figure S9 Alignment of short neuropeptide F (sNPF) peptides from *C. suppressalis* (Cs), *B. mori* (Bm), *A. mellifera* (Am), and *D. melanogaster* (Dm). Identities are highlighted in dark red, and similarities are indicated by gray.

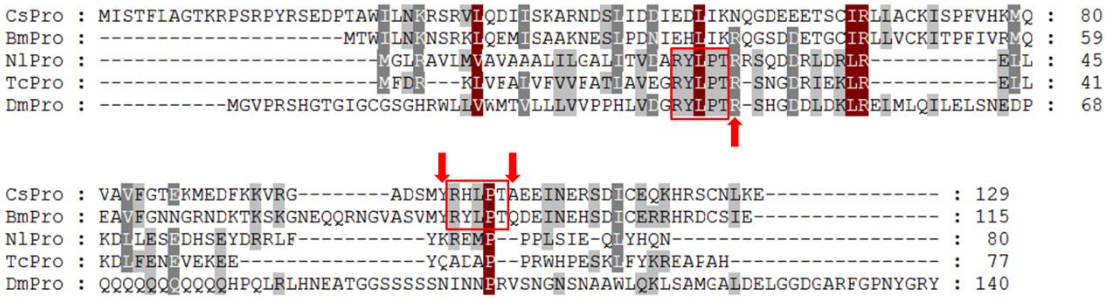

Figure S10 Protein alignment of proctolin (Pro) precursor sequences from *C. suppressalis* (Cs), *B. mori* (Bm), *N. lugens* (Nl), *T. castaneum* (Tc), and *D. melanogaster* (Dm). Identities are highlighted in dark red, and similarities are indicated by gray. The solid boxes indicate the mature peptides. The arrows indicate potential cleavage sites.
